# Isoenzyme characterization of *Leishmania infantum* toward checking the antioxidant activity of superoxide dismutase and glutathione peroxidase

**DOI:** 10.1186/s12879-024-09069-7

**Published:** 2024-02-15

**Authors:** Mostafa Alishvandi¹, Somayeh Bahrami, Sajad Rashidi, Gholamreza Hatam

**Affiliations:** 1 ¹Department of Parasitology and Mycology, Shiraz University of Medical Sciences, Shiraz, Iran; 2 Shahid Chamran University of Ahwaz, Ahvaz, Iran; 3 Molecular and Medicine Research Center, Khomein University of Medical Sciences, Khomein, Iran; 4 Department of Medical Laboratory Sciences, Khomein University of Medical Sciences, Khomein, Iran; 5 Basic Sciences in Infectious Diseases Research Center, Shiraz University of Medical Sciences, Shiraz, Iran

**Keywords:** *Leishmania Infantum*, Isoenzyme electrophoresis, Antioxidant enzymes

## Abstract

**Background:**

*Leishmania infantum* is the major causative agent of visceral leishmaniasis in Mediterranean regions. Isoenzyme electrophoresis (IE), as a biochemical technique, is applied in the characterization of *Leishmania* species. The current study attempted to investigate the isoenzyme patterns of logarithmic and stationary promastigotes and axenic amastigotes (amastigote-like) of *L. infantum* using IE. The antioxidant activity of superoxide dismutase (SOD) and glutathione peroxidase (GPX) was also checked in the aforementioned forms.

**Method:**

After *L. infantum* cultivation and obtaining logarithmic and stationary promastigotes, axenic amastigotes were achieved by incubation of stationary promastigotes at 37 °C for 48 h. The lysate samples were prepared and examined for six enzymatic systems including glucose-6-phosphate dehydrogenase (G6PD), nucleoside hydrolase 1 (NH1), malate dehydrogenase (MDH), glucose-phosphate isomerase (GPI), malic enzyme (ME), and phosphoglucomutase (PGM). Additionally, the antioxidant activity of SOD and GPX was measured.

**Results:**

GPI, MDH, NH1, and G6PD enzymatic systems represented different patterns in logarithmic and stationary promastigotes and axenic amastigotes of *L. infantum*. PGM and ME showed similar patterns in the aforementioned forms of parasite. The highest level of SOD activity was determined in the axenic amastigote form and GPX activity was not detected in different forms of *L. infantum*.

**Conclusion:**

The characterization of leishmanial-isoenzyme patterns and the measurement of antioxidant activity of crucial antioxidant enzymes, including SOD and GPX, might reveal more information in the biology, pathogenicity, and metabolic pathways of *Leishmania* parasites and consequently drive to designing novel therapeutic strategies in leishmaniasis treatment.

**Supplementary Information:**

The online version contains supplementary material available at 10.1186/s12879-024-09069-7.

## Introduction

*Leishmania* parasites are the causative agents of a group of infectious diseases called leishmaniasis. The clinical manifestations of leishmaniasis are varied in different forms of disease (Cutaneous Leishmaniasis (CL), Mucocutaneous Leishmaniasis (MCL), and Visceral Leishmaniasis (VL)) [1]. *Leishmania infantum* is the causative agent of VL in different animal reservoirs and hosts (humans) in the Mediterranean area [2].

After the entrance of promastigotes (the form of parasite in vectors, phlebotomine sandflies) into the infected host, the macrophages engulf the promastigotes and change them to intracellular pathogenic forms (amastigote) [3]. In in vitro conditions (incubating at 37 °C and 5% CO_2_), cultivated promastigotes in the stationary stage can produce the axenic amastigotes (amastigote-like) [4].

The molecular biology, survival and replication, pathogenicity, and metabolism of *Leishmania* parasites are relevant to the different genes, proteins and enzymes in parasite forms (promastigote and amastigote) and secretion [5]. Different techniques including proteomics and genomics have been successfully developed for the recognition of such crucial biomarkers in different parasites including *Leishmania* [6–8]. In addition, isoenzyme tools can facilitate the characterization of enzyme and isoenzyme patterns of such parasites [9].

This study attempted to characterize the enzymatic systems including glucose-6-phosphate dehydrogenase (G6PD), nucleoside hydrolase 1 (NH 1), malate dehydrogenase (MDH), glucose-phosphate isomerase (GPI), malic enzyme (ME) and phosphoglucomutase (PGM) in logarithmic and stationary promastigotes, and also in axenic amastigotes of *L. infantum* using Isoenzyme Electrophoresis (IE) technique. All these enzymes are involved in pivotal metabolic processes during parasitic (leishmaniasis) infections. The information from real amastigotes (obtained from mammalian cells) will be more informative compared to the axenic form. However, the achievement of pure amastigotes from infected-host cells is difficult in IE approaches since the released enzymes from residual debride cells may interfere with the parasitic (amastigote) enzymes. Since the deletion of such unwanted released enzymes is approximately impossible in such approaches, the axenic amastigotes were applied instead of real amastigotes in this study. Several studies confirmed the high similarity between proteome and genome (and probably enzymatic) profiles of real amastigotes and axenic forms.

The host immune system increases the antioxidants against *Leishmania* parasites for the management of leishmaniasis infection. SODs production is a defensive mechanism employed by *Leishmania* parasites dealing with the host immune responses [10–12]. On the other hand, glutathione biosynthesis and arginase activity are crucial mechanisms in the biology of *Leishmania* parasites. For instance, the inhibition of glutathione biosynthesis and arginase activity has been underlined as possible strategies in the treatment of leishmaniasis [10]. Accordingly, the antioxidant activity of superoxide dismutase (SOD) and glutathione peroxidase (GPX) was also measured in logarithmic and stationary promastigotes, and also in axenic amastigotes of *L. infantum*.

Overall, the characterization of leishmanial-isoenzymes and the measurement of antioxidant activity of crucial antioxidant enzymes, including SOD and GPX, might reveal more information in the biology, pathogenicity, and metabolic pathways of *Leishmania* parasites and consequently drive to designing of novel therapeutic strategies in leishmaniasis treatment [13, 14].

## Method

*L. infantum* strain (MCAN/07/MOHEB/AH-1) was provided by the Department of Parasitology and Mycology, Shiraz University of Medical Sciences, Shiraz, Iran. In this study, we used the standard strain of the parasite, thus, the ethical approval was not applicable.

### Parasite culture

#### Logarithmic and stationary promastigotes

Briefly, *L. infantum* promastigotes were propagated in RPMI-1640 (Shelmax Company) supplemented with 15% (v/v) heat-inactivated fetal calf serum (FCS), 100 U/mL penicillin and 100 µL/mL streptomycin at 25 °C. Then, promastigotes were sub-cultured for 3 and 7 days to produce logarithmic and stationary phases, respectively [3].

#### Axenic amastigotes

10^6^ promastigotes (in stationary phase) were dispensed in each well of a cell culture plate and incubated at 37 °C (5% CO_2_, pH = 5) for 48 h to obtain axenic amastigotes. The viability of axenic amastigotes was checked using trypan blue (TB) staining [4].

### Checking the morphological structure (using Giemsa staining) and viability of axenic amastigotes (using TB)

Giemsa staining and microscopic methods were used to check the morphological structure of axenic amastigotes. TB, as a vital dye for checking the cell viability, penetrates dead cells and dead cells are stained. In the TB experiment, 10 µL of medium containing 10^4^ axenic amastigotes was added to 10 µL of TB, and the viability was immediately checked using the Neubauer hemocytometer and microscopic method [15]. The exposed axenic amastigotes to the methanol were considered a positive control.

### Biochemical characterization

#### Enzyme stabilizer preparation

7.445 g ethylene diamine tetra acetic acid (EDTA) was dissolved in 50 mL of Double-distilled (DD) water to solve with 200 mM density. The final total volume was 100 ml by adding it with deionized water and aliquoted into 10 ml tubes. Then, 1 ml of this solution was added to 0.0308 g dithiothreitol. 0.0262 g E-aminocaproic acid was added to the solution to achieve 200 mM density. 1 ml of enzyme stabilizer solution was added in 10 ml DD water to produce 2 mM density.

#### Lysate’s preparation

One hundred mL culture medium containing ten million parasites/mL of each form (logarithmic and stationary promastigote, and axenic amastigote) was counted and centrifuged at 2000× g for 20 min at 4 °C. The supernatants were removed and the obtained pellets were washed three times with cold PBS at 1250× g for 10 min at 4 °C. After adding an equal volume of the enzyme stabilizer to the pellets, the contents were mixed. Then, the freeze-thaw procedure (at -196 °C and 4 °C) was performed five times. The obtained extracts were centrifuged at 18,000× g for 60 min at 4 °C. Finally, supernatants were stored at -70 °C for IE experiments [9].

#### Gel preparation

Separating gel 7% (10 ml) was produced by mixing all reagents of AcrylamidBis, resolving gel buffer, and deionized water. 250 µL of 10% ammonium persulfate (APS) was added to the solution, and 25 µL of tetramethylethylenediamine (TEMED) was added and gently mixed. Then, a thin layer of deionized water was added. The gel was immediately poured using a pipette into a glass plate (2/3 of the whole volume). The gel was allowed to be set for 40 min at RT (room temperature). 4% of the stacking gel (10 ml) was prepared by mixing all of the reagents including AcrylamidBis, staking gel buffer, and deionized water. 250 µL of 10% APS was added to the solution. Then, 25 µL of TEMED was added and gently mixed. The gel was piped onto the separating gel. In the next step, the appropriate comb was immediately inserted into the gel. The stacking gel was allowed to polymerize for 40 min at RT.

#### Samples insertion

The plates and gel were moved into the tank compartment of the electrophoresis chamber. The running buffer was added into the chambers of the apparatus. The samples were prepared by mixing 10 µL of each sample with 10 µL of loading buffer. The mixture was loaded into the wells using a microsyringe.

#### IE

IE was conducted for each sample (logarithmic and stationary promastigote, and axenic amastigotes) using a discontinuous polyacrylamide gel electrophoresis system. *L. major* strain (MCAN/IR/97/LON490) was used as the control (with distinct isoenzyme patterns) in the IE assay. All samples were examined for six enzymatic systems including G6PD (E.C. 1.1.1.49), NH 1 (E.C.3.2.2.1), MDH (E.C. 1.1.1.37), GPI (E.C.5.3.1.9), ME (E.C.1.1.1.40), and PGM (E.2.7.51). Approximately 2.5 mA power for each well was adjusted for 1 h until the marker dyes reached the bottom edge of the separating gel. After completing IE, the separating gel gently was put into the clean glass container for the next step (staining). Specific coenzymes and substrates were used for each enzymatic system in IE assays. Thus, the presence of enzymatic activities like oxidase, could not interfere with IE assays.

#### Enzymes staining

After IE, the prepared specific reagent for each enzyme system was poured onto the gel so that it covered all the gel, and then the gel was incubated at 37 °C for 25–35 min for G6PD, GPI, MDH, ME, NH 1 and PGM.

#### Staining reaction

The relative migration distance or relative factor (RF) graphically was identified for the all bands in the samples. The migration distance was measured from the top of the resolving gel to each band and to the dye front using a ruler. RF value was measured by the use of this equation: RF = migration distance of the protein band/migration distance of the dye front.

### Determination of the activity of SOD and GPX

SOD activity is determined according to the creation of superoxide radicals produced by xanthine and xanthine oxidase, which react with 2-(4-iodophenyl)-3-(4-nitrophenol)-5- phenyltetrazolium chloride to compose a red formazan dye. Using the manufacturer’s instruction (Ransod®- Randox Lab, Antrim, UK), SOD activity was calculated based on the obtained absorbance at 505 nm.

GPX can catalyze the oxidation of glutathione using cumene hydroperoxide. In the presence of nicotinamide adenine dinucleotide phosphate (NADPH) and glutathione reductase, the oxidized glutathione is directly converted to the reduced form with concomitant oxidation of NADPH to NADP^+^. Using the manufacturer’s instruction (Ransod®- Randox Lab, Antrim, UK), GPX activity was measured based on the obtained absorbance at 340 nm. The antioxidant activity of SOD and GPX was reported as U/ml.

### Statistical analysis

One-way ANOVA and SPSS 17 (SPSS Inc., Chicago, IL, USA) were used for statistical analysis in this study. Regarding all obtained data, a P-value < 0.05 was considered statistically significant.

## Results

### Checking the morphological structure and viability of axenic amastigotes

**Fig. 1:**
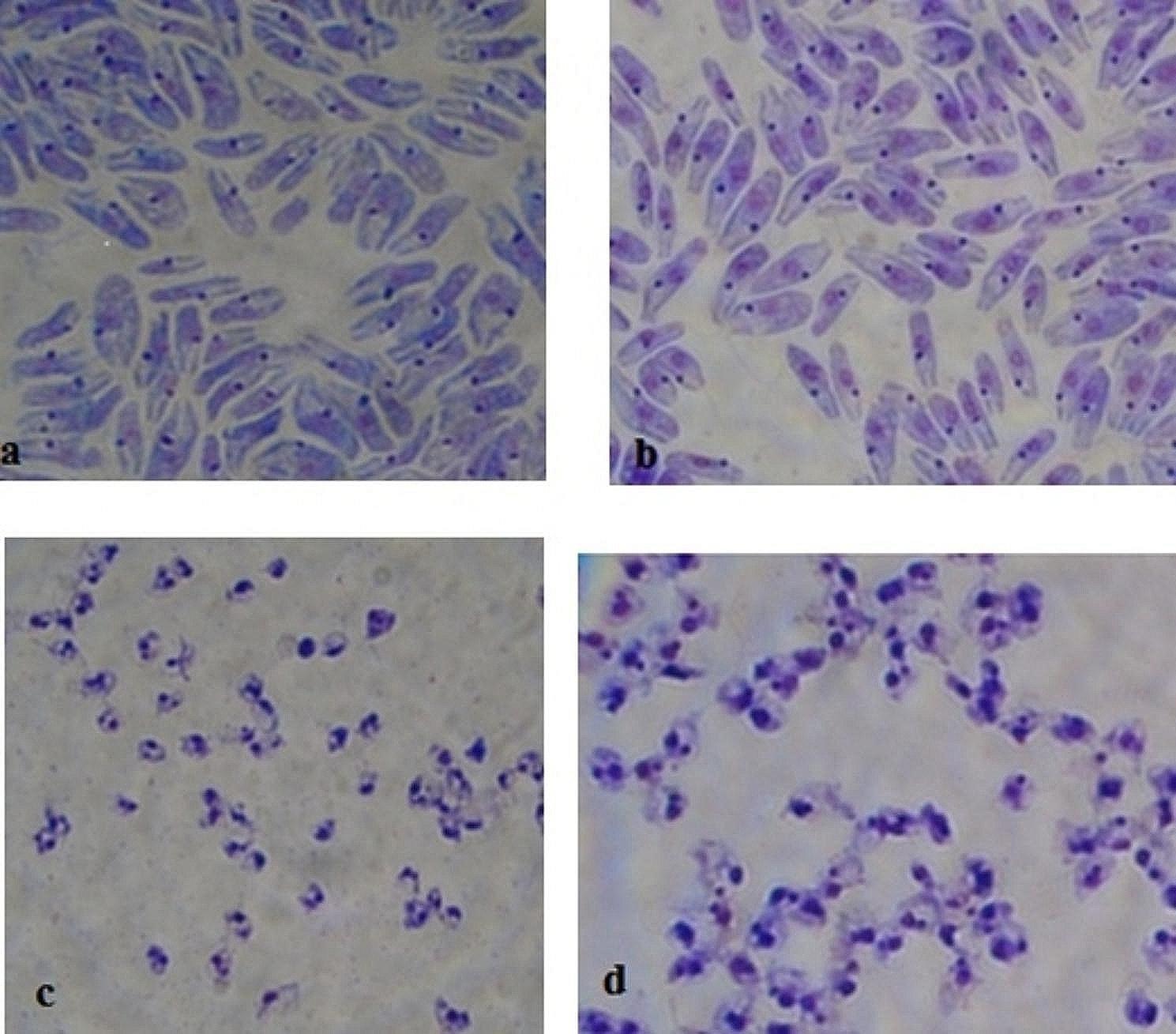
*L. infantum*: (**a**) logarithmic promastigotes, (**b**) stationary promastigotes, (**c**) axenic amastigotes after 24 h, (**d**) axenic amastigotes after 48 h (1000 X)

A nucleus, a rod-like kinetoplast, and a homogeneous cytoplasm were shown in axenic amastigotes due to the Giemsa staining and microscopic investigations. The size of the axenic amastigote was approximately 2–4 µ and the anterior flagellum was not seen in such intracellular form (Fig. 1). The viability percent of promastigotes in the logarithmic phase was approximately 100% using the TB and microscopic method. In axenic amastigotes, this ratio was determined > 90%. As expected, in the stationary phase of promastigote the rate of dead cells was increased (approximately 60%).

### Biochemical characterization

#### IE

**Fig. 2:**
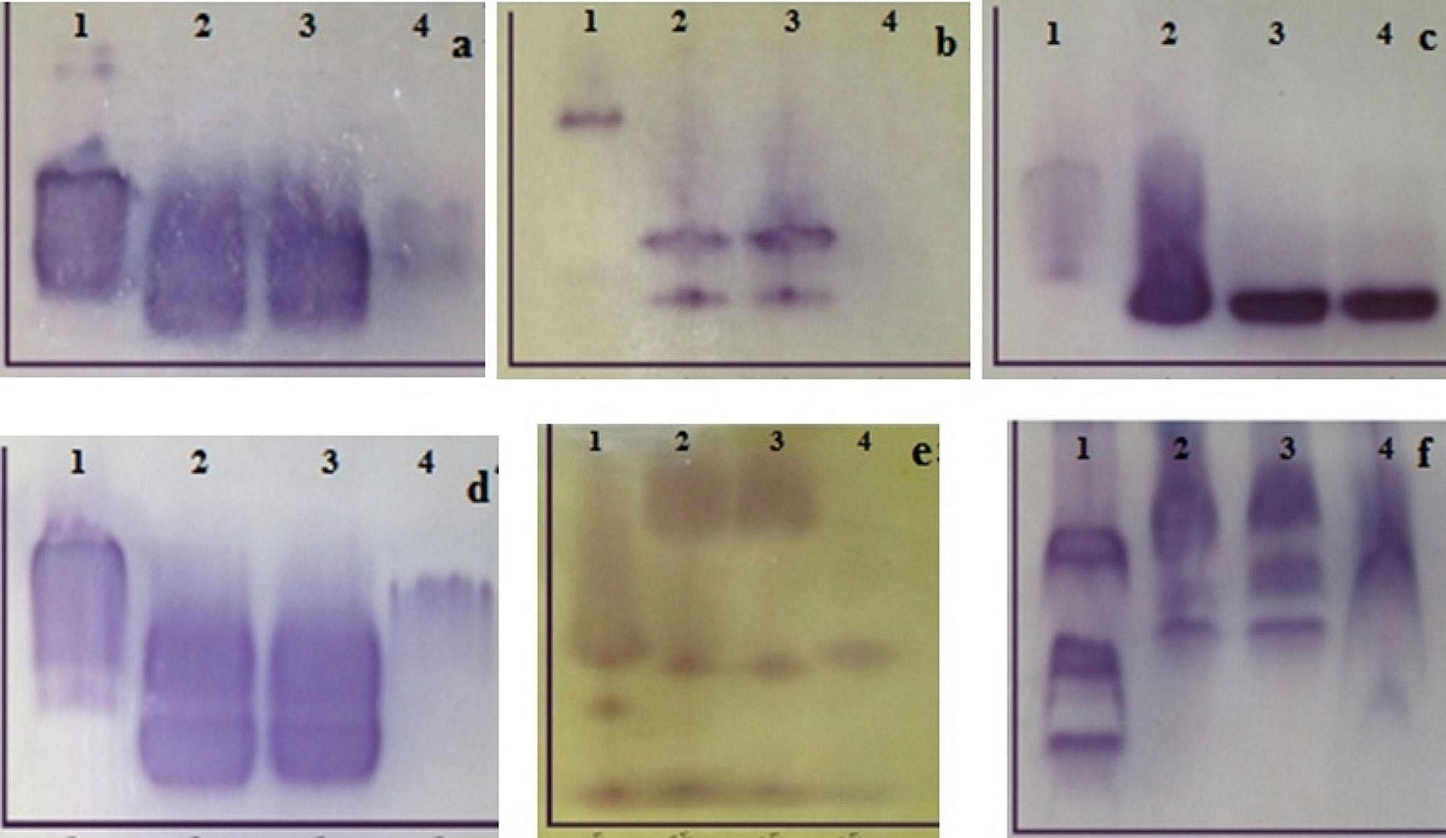
Enzymatic systems of *L. major* (control) and *L. infantum*: (**a**) GPI, (**b**) ME, (**c**) PGM, (**d**) G6PD, (**e**) NH I, (**f**) MDH. Lane 1- logarithmic promastigotes of *L. major*, lane 2- logarithmic promastigotes of *L. infantum*, lane 3- stationary promastigotes of *L. infantum*, lane 4- axenic amastigotes of *L. infantum*

**Fig. 3:**
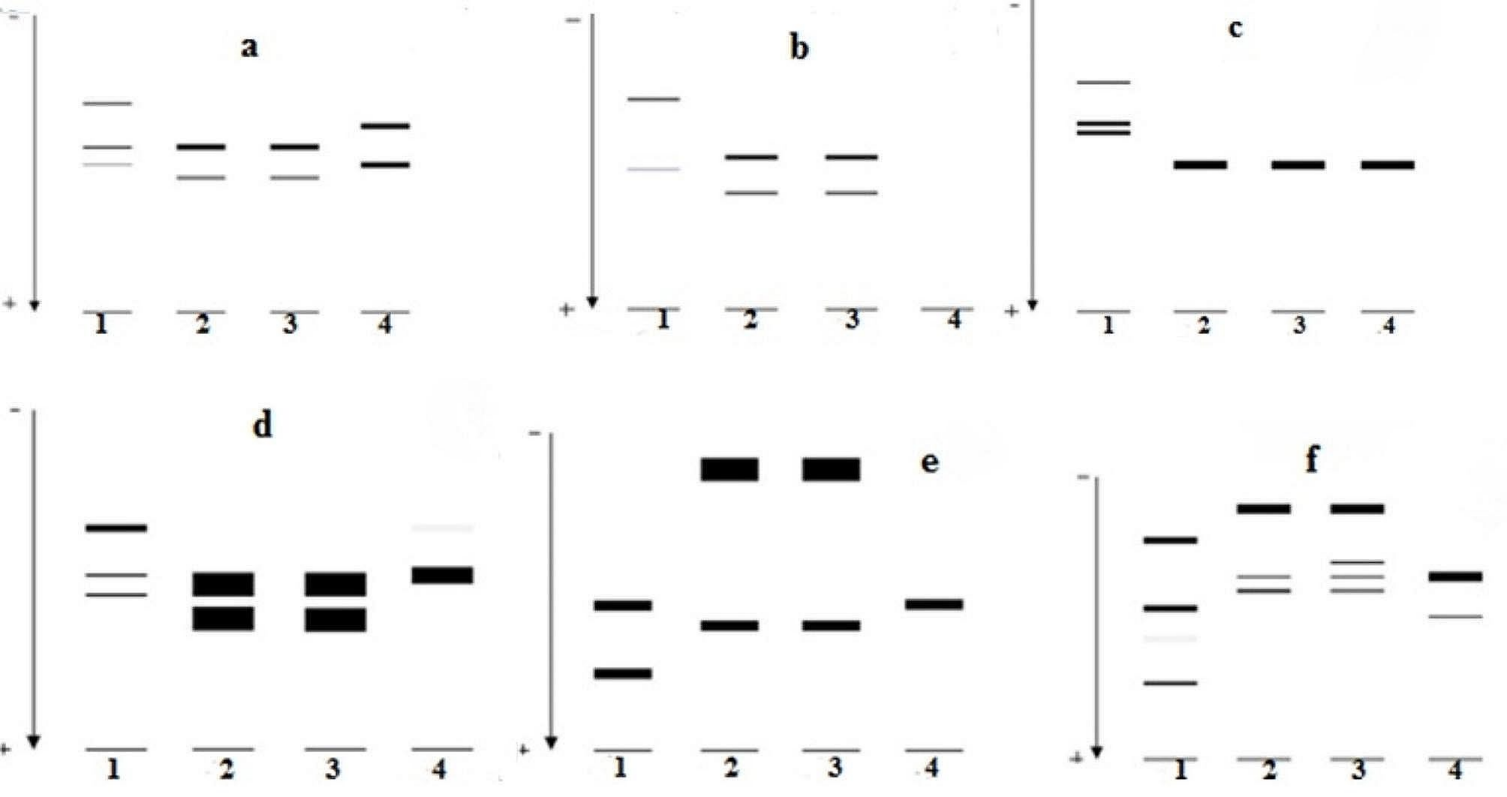
Diagrammatic representation of enzymatic systems in *L. major* (control) and *L. infantum*: (**a**) GPI, (**b**) ME, (**c**) PGM, (**d**) G6PD, (**e**) NH I, (**f**) MDH. Lane 1- logarithmic promastigotes of *L. major*, lane 2- logarithmic promastigotes of *L. infantum*, lane 3- stationary promastigotes of *L. infantum*, lane 4- axenic amastigotes of *L. infantum*

Six enzymatic systems were evaluated in logarithmic and stationary promastigotes and axenic amastigotes of *L. infantum* using IE and polyacrylamide gel (Figs. 2 and 3). GPI, MDH, NH 1, and G6PD patterns were different in the aforementioned forms of *L. infantum* parasite. However, PGM and ME represented similar enzymatic patterns in logarithmic and stationary promastigotes and axenic amastigotes of *L. infantum*. Migration bands were not detected in axenic amastigote form in the ME enzymatic system.

### Determination of the activity of SOD and GPX

The antioxidant enzyme activity of SOD and GPX was determined in logarithmic and stationary promastigotes and axenic amastigotes of *L. infantum*. SOD showed the highest level of activity in axenic amastigotes. The antioxidant activity of SOD was calculated as 27.33 ± 11.5, 32 ± 12.76, and 98.33 ± 25.7 U/ml in logarithmic and stationary promastigotes, and axenic amastigotes, respectively. GPX activity was not detected in the aforementioned forms of *L. infantum*.

## Discussion

Different molecular techniques including genomics have been successfully used for the identification of species of *Leishmania* parasites. In combination with genomics, proteomics tools can further provide valuable information about the biology and pathogenesis of such parasites [6–8]. On the other side, biochemical techniques such as IE can exert an important role in the characterization of enzymatic patterns and metabolic events that occurred in different forms (life stages) of *Leishmania* spp [9].. Although IE approaches are time-consuming techniques and have been less noticed recently, the integration of such tools with data inferred from genomics and proteomics can drive to reveal more information concerning the pathobiology of pathogens such as *Leishmania* parasites in different aspects including metabolism and immunometabolism.

There are several studies regarding the characterization of *Leishmania* spp. in the literature. For instance, the use of the IE technique and the identification of isoenzyme patterns have exerted important roles in the determination of *L. tropica* and *L. infantum* as the major causative agents of the lupoid form of cutaneous leishmaniasis and VL, respectively, in Iran [16–18]. On the other side, such techniques indicated the presence of six species and 21 zymodemes of *Leishmania* parasites [19]. Another study has shown similar patterns of PGM, 6PGD, MD, and NH 2 enzymatic systems in different forms of *L. tropica*. However, G6PD, GPI, NH I, and ME were introduced as enzymatic systems with diverse patterns [20]. The correlations of polymorphism in *Leishmania* isoenzymes and clinical symptoms and geographical distribution of leishmaniasis have been also highlighted [18, 19]. Despite this information, new published data regarding the isoenzyme pattern of *L. infantum* is scarce.

The preparation of samples with a high quality is a crucial issue in IE approaches. Since the high rate of dead parasites (cells) in samples can lead to the achievement of unreliable results, checking the cell viability is necessary before doing IE experiments. As mentioned in the results, the rate of viability in the logarithmic phase of promastigotes and axenic amastigotes was acceptable for conducting IE experiments. However, the isozyme patterns probably have been affected by the inevitable increase in the rate of dead promastigotes in the stationary phase.

In the present study, six enzymatic systems were evaluated in logarithmic and stationary promastigotes and axenic amastigotes of *L. infantum*. The results indicated different patterns of GPI, MDH, NH 1, and G6PD enzymatic systems in logarithmic and stationary promastigotes, and axenic amastigotes of *L. infantum*. Thus, it can be concluded that such enzymatic systems might be more efficient for the characterization of different *Leishmania* species. PGM and ME systems showed similar patterns in different forms of parasite.

The presence of the ME system was not confirmed in axenic amastigotes. Although all of the abovementioned enzymes are involved in pivotal metabolic processes and cell responses (such as immune responses) during parasitic (leishmaniasis) infections, each stage of the *Leishmania* parasites (including logarithmic and stationary promastigotes, axenic amastigotes) probably recruits distinct strategy to growth and replicate and also enter into the infected host cells, so the enzyme levels and activities could be variable in each parasite stage.

Infected macrophages apply different mechanisms including the production of reactive nitrogen species (RNS) and reactive oxygen species (ROS), acidification of phagolysosomes and digestion by hydrolytic enzymes against the presence of *Leishmania* parasites [21–24]. In contrast, *Leishmania* parasites develop some strategies containing the detoxification of RNS and ROS for adaption to the macrophage environment [25]. Moreover, the production of peroxiredoxins, catalases, SODs, and glutathione S-transferase/GPX coupled with glutathione reductase has been indicated as the enzymatic defensive strategy against ROS [13]. According to this information, the antioxidant activity of SOD and GPX enzymes was measured in logarithmic and stationary promastigotes and axenic amastigotes of *L. infantum* in this study. Our findings showed the highest level of SOD activity in axenic amastigotes of *L. infantum*. Since the amastigote is the intracellular form of *Leishmania* parasites in macrophages, expressing the highest level of SOD activity in axenic amastigotes might further confirm the role of SODs in the protection and adaption of amastigotes against macrophage defense mechanisms. In addition, the stationary phase of *Leishmania* promastigotes is considered the invasive form of parasite against host cells. Since the host immune system increases antioxidants against *Leishmania* parasites, obtaining an appropriate level of SOD activity in the stationary phase in comparison to the logarithmic phase might further elucidate the potential function of SODs in the induction of pathogenesis process of *Leishmania* parasites.

As mentioned, GPX is one of the major defense factors against ROS in the mammalian cell against pathogens [26, 27]. Although glutathione biosynthesis is considered an important mechanism in the biology of *Leishmania* parasites, information regarding GPX is scarce in such parasites. According to the obtained data in this study, GPX activity was not detected in the logarithmic and stationary promastigotes and axenic amastigotes of *L. infantum*. As a probable hypothesis, the absence of oxidative pressure in the culture forms of *L. infantum* parasites might be related to undetectable GPX activity. However, the GPX activity has been measured in cultured *L. major* and *L. tropica* metacyclic and procyclic promastigotes [28]. Since other different factors such as protein concentration are involved in the evaluation of enzyme activity in IE approaches, more investigations are needed to corroborate GPX activity in *Leishmania* parasites.

## Conclusion

The characterization of leishmanial-isoenzyme patterns and the measurement of antioxidant activity of crucial antioxidant enzymes, including SOD and GPX, might reveal more information in the biology, pathogenicity, and metabolic pathways of *Leishmania* parasites and consequently drive to designing novel therapeutic strategies in leishmaniasis treatment.

### Electronic supplementary material

Below is the link to the electronic supplementary material.

Supplementary Material 1

## Data Availability

All data generated or analysed during this study are included in this published article.
