# Supplementary material for: Isoenzyme characterization of Leishmania infantum toward checking the antioxidant activity of superoxide dismutase and glutathione peroxidase
Source: BMC Infect Dis. 2024 Feb 15;24:208. doi: 10.1186/s12879-024-09069-7 (PMC10870465; doi:10.1186/s12879-024-09069-7)

In-house Editorial Comments

We note that some of the images are heavily cropped. We ask that as full as possible length gels and blots are included in the Supplementary Information file. These images should be the original, unprocessed versions.

Response: we represented here these pictures as In-house Editor requested the origin pictures.

Please note that it is not required to add them in article add supplementary data.

Thanks


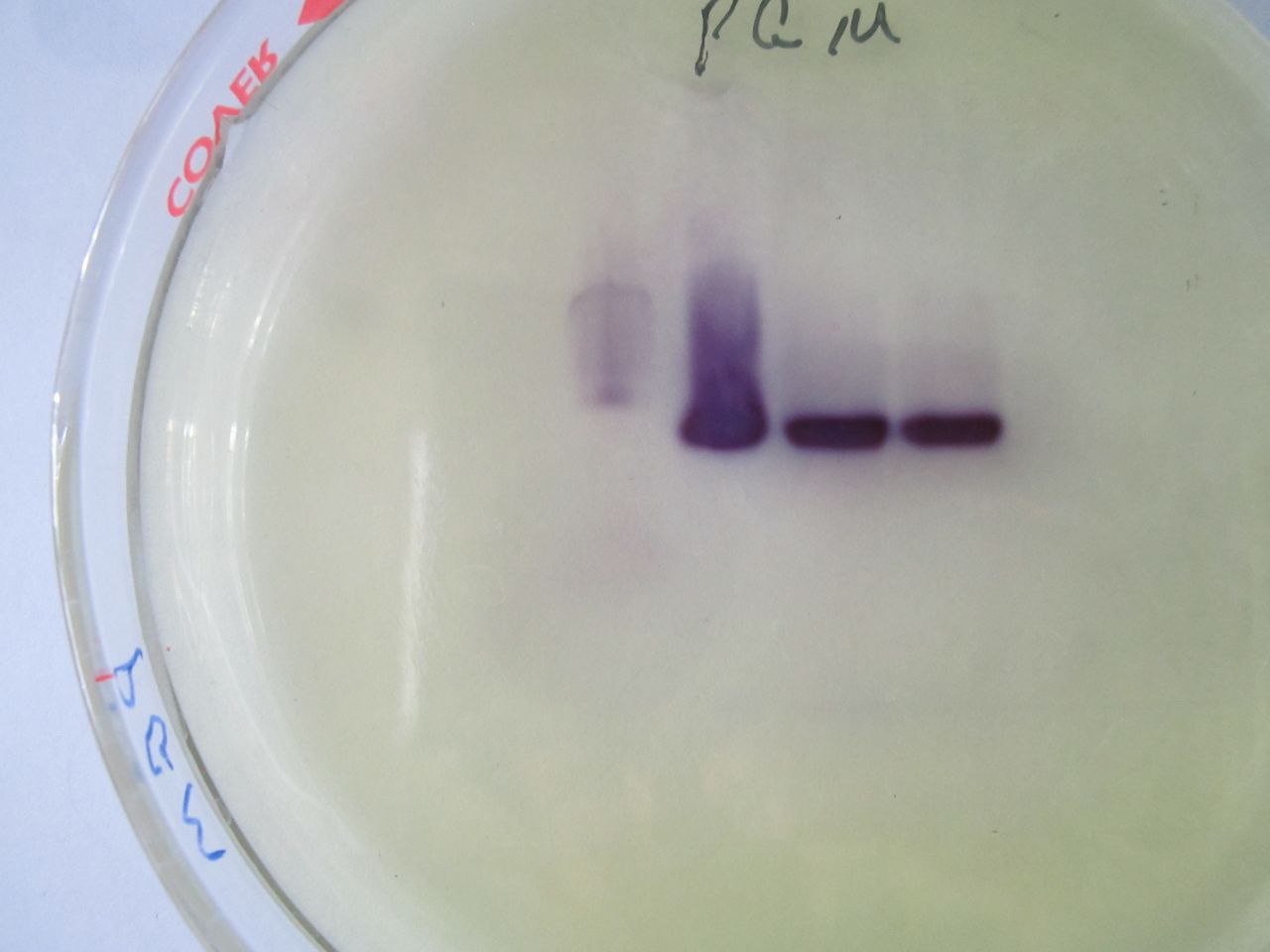


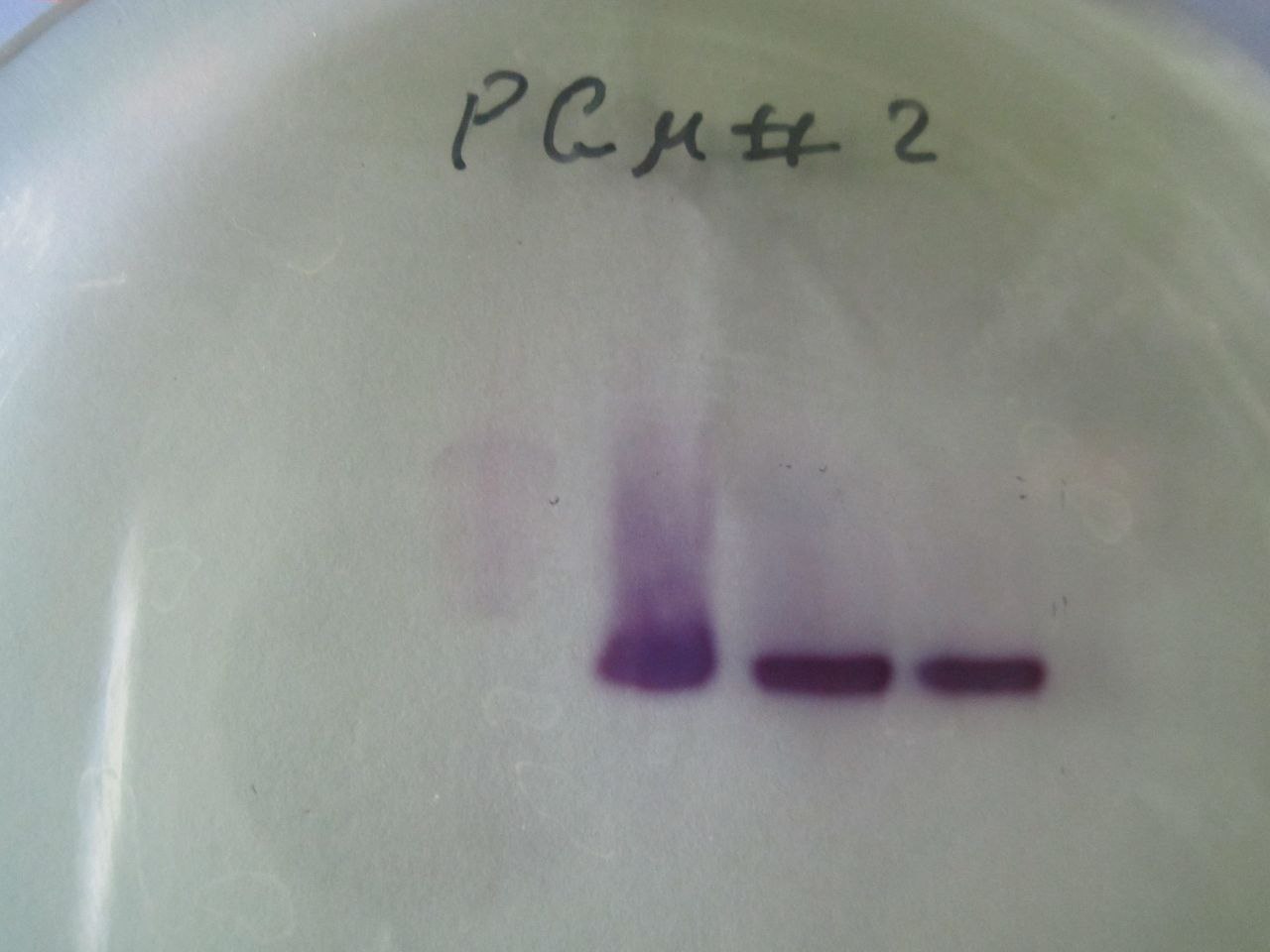


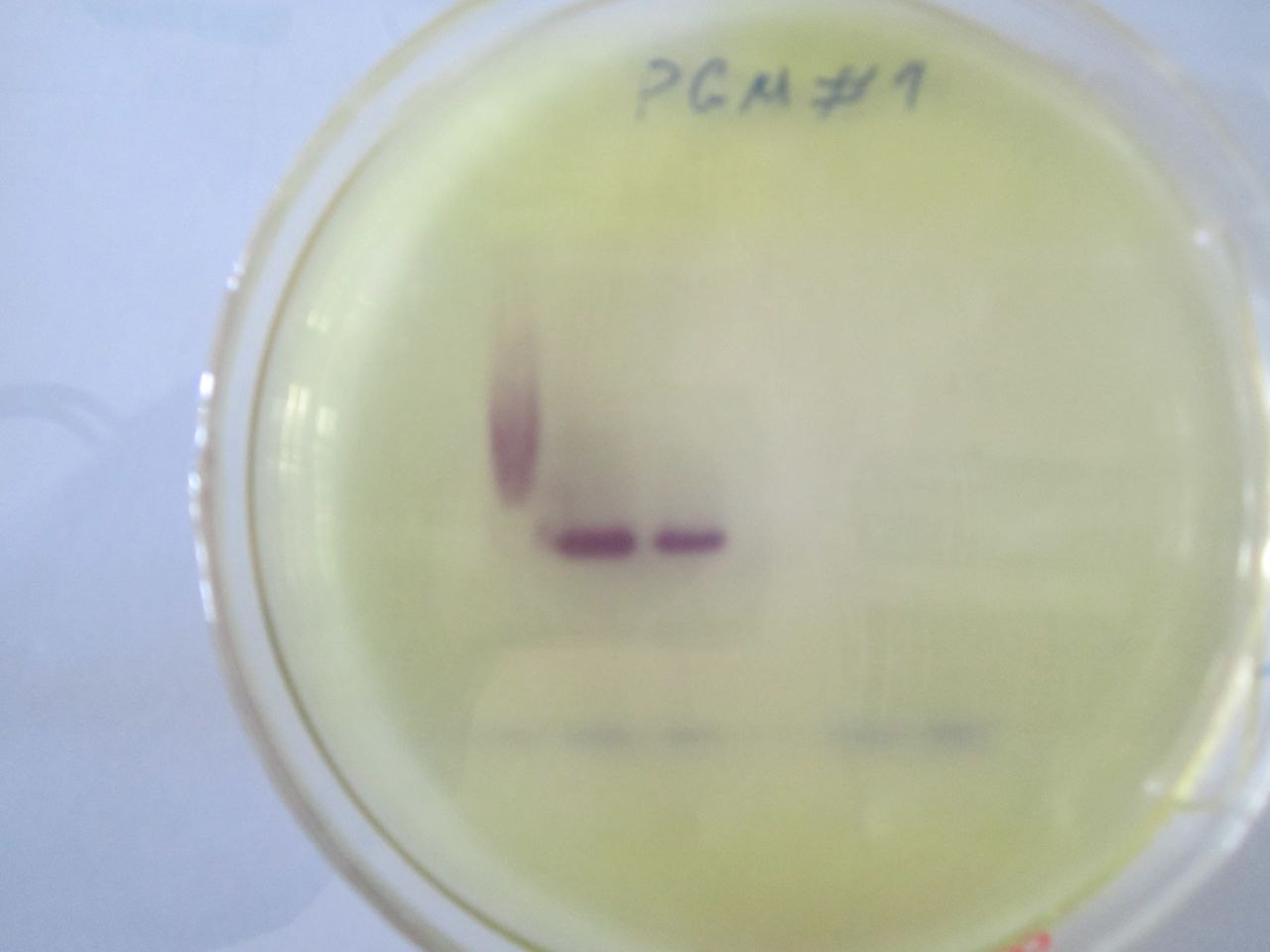


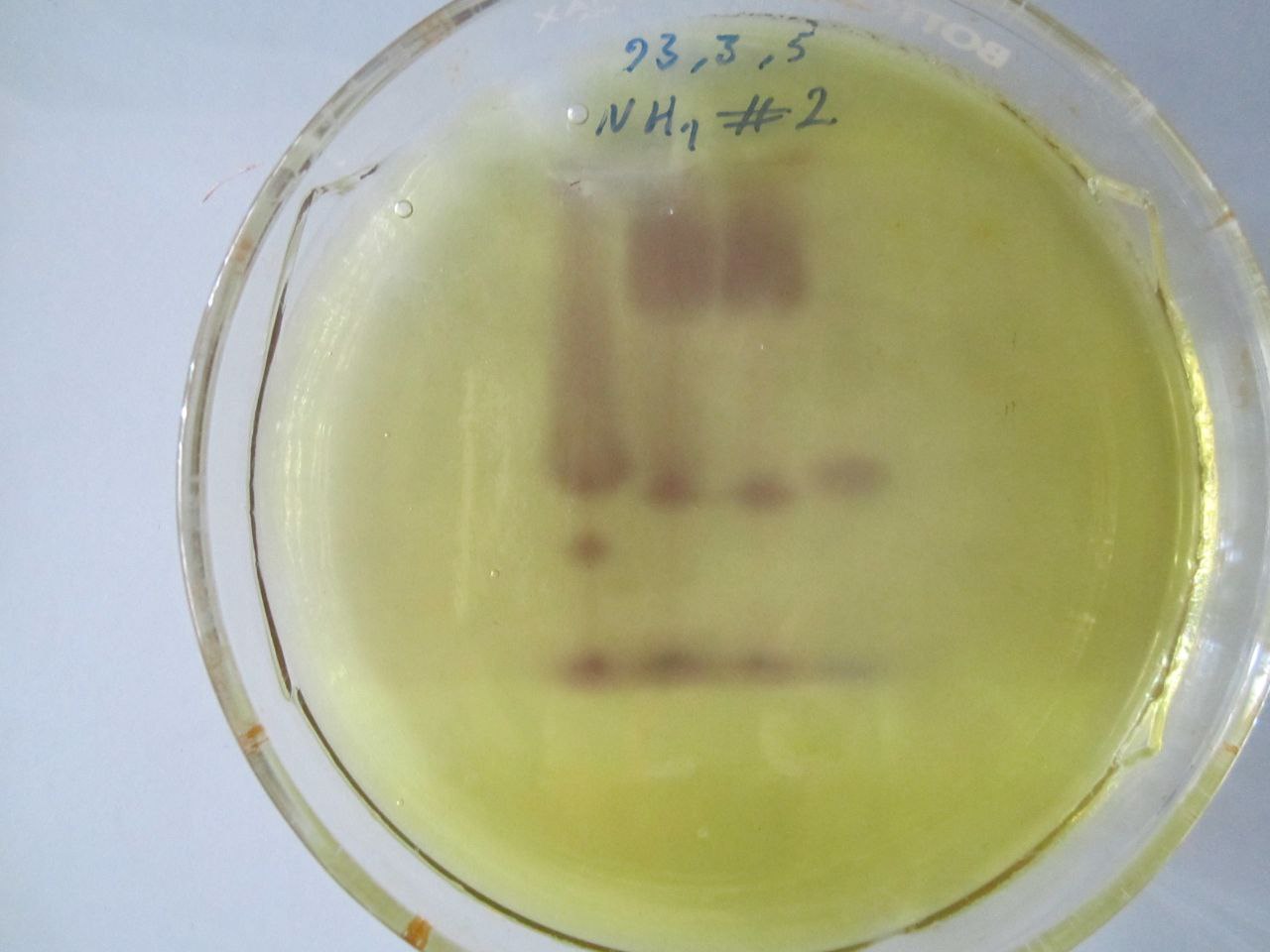


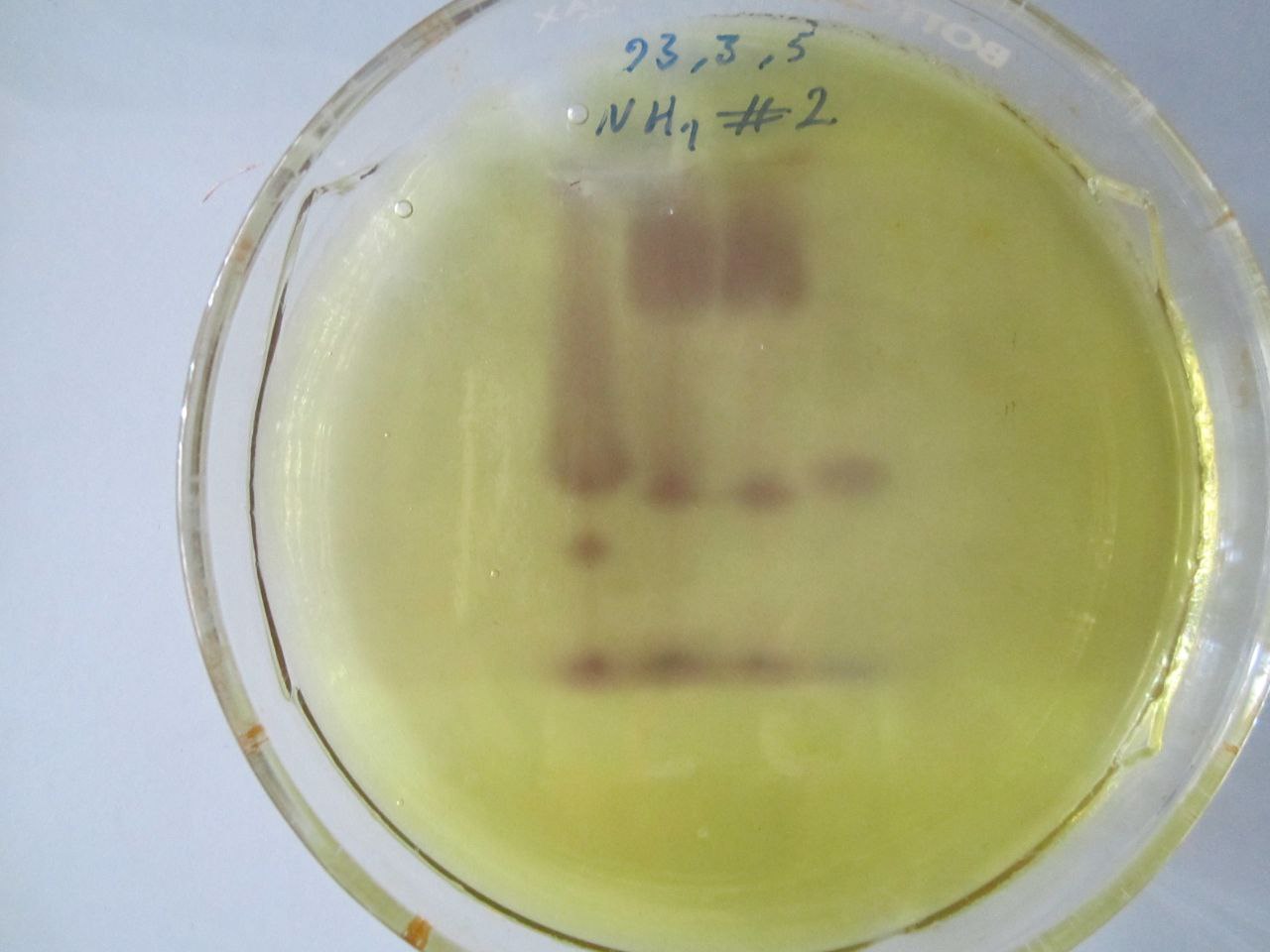


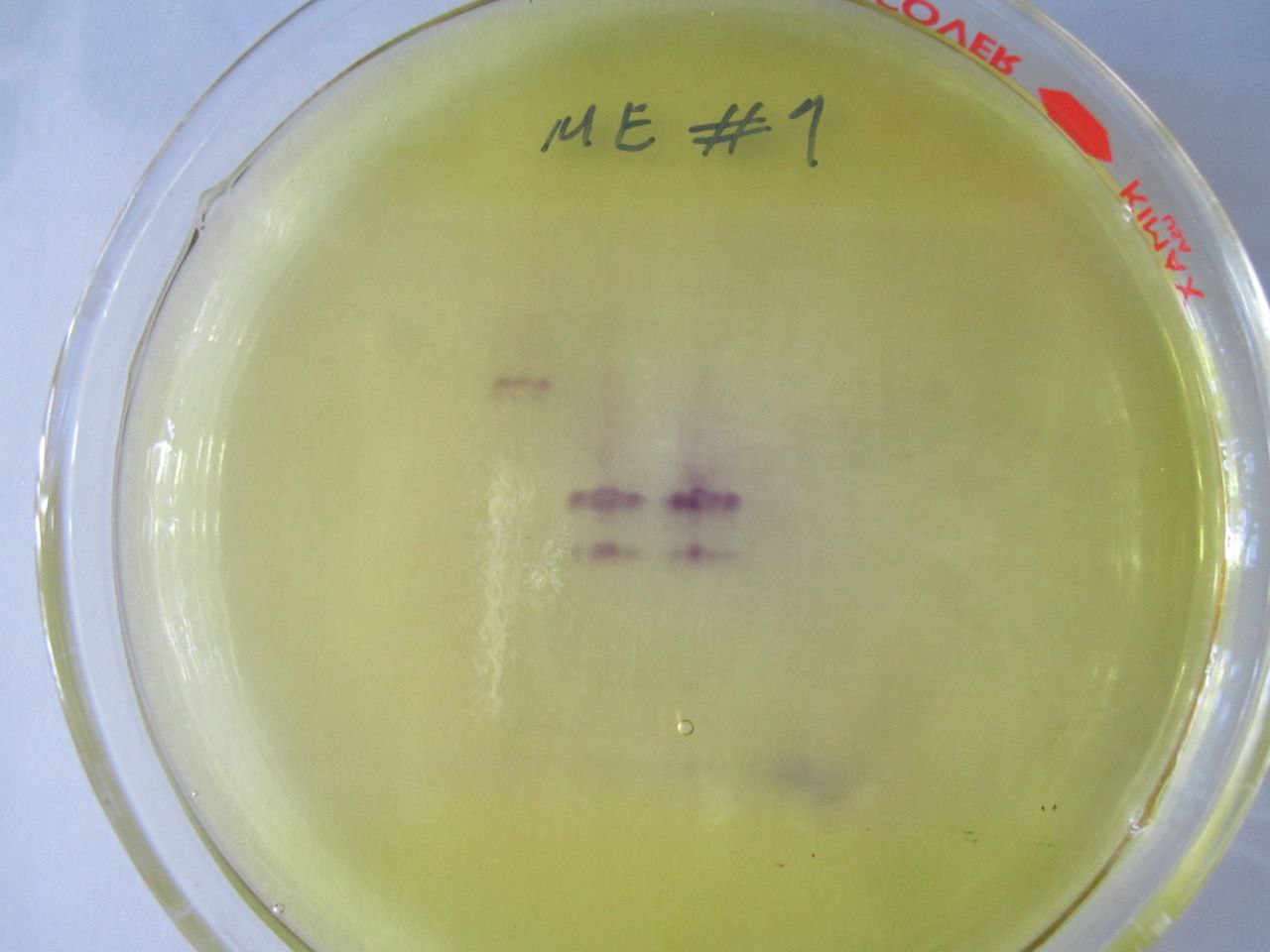


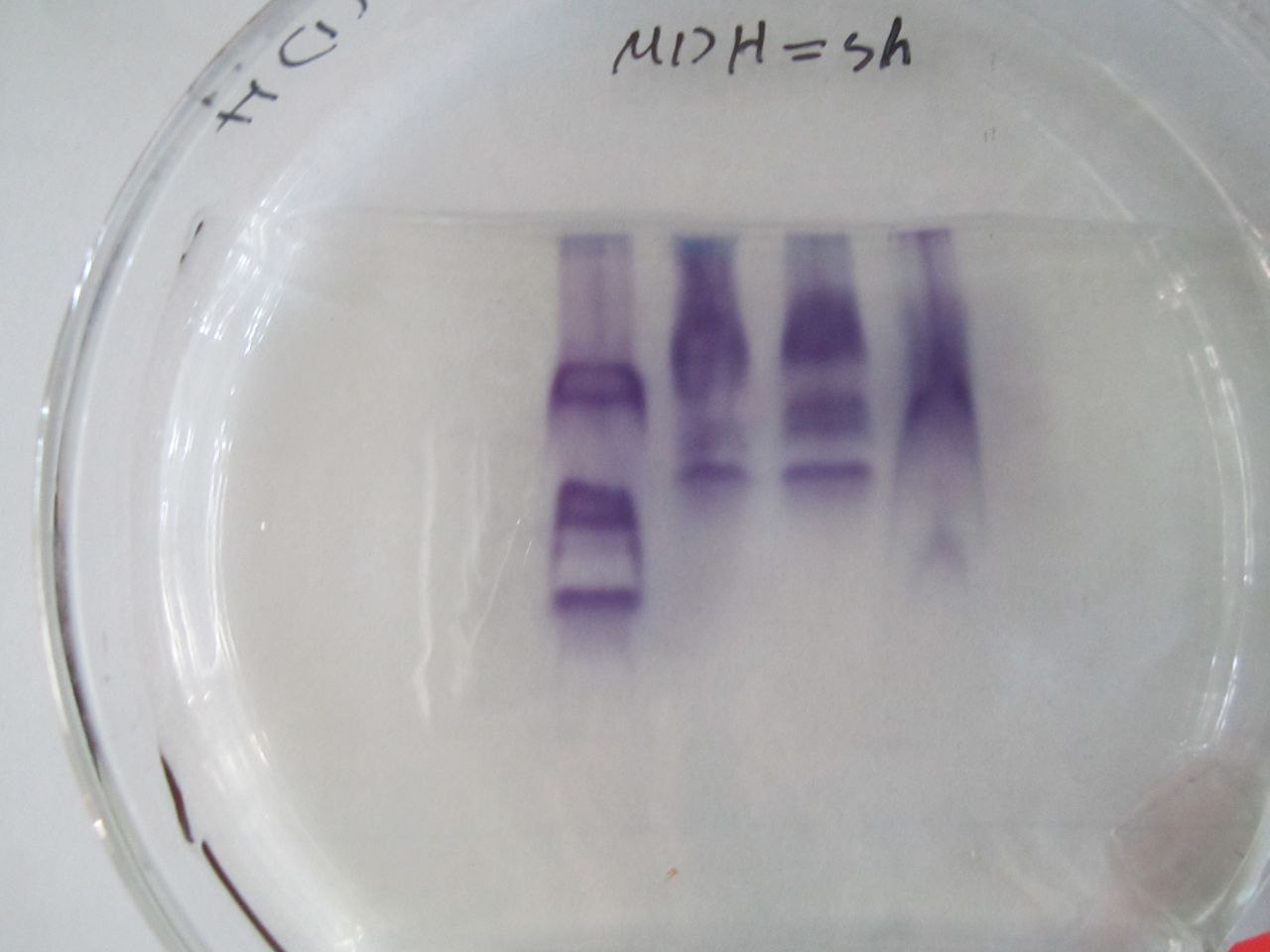


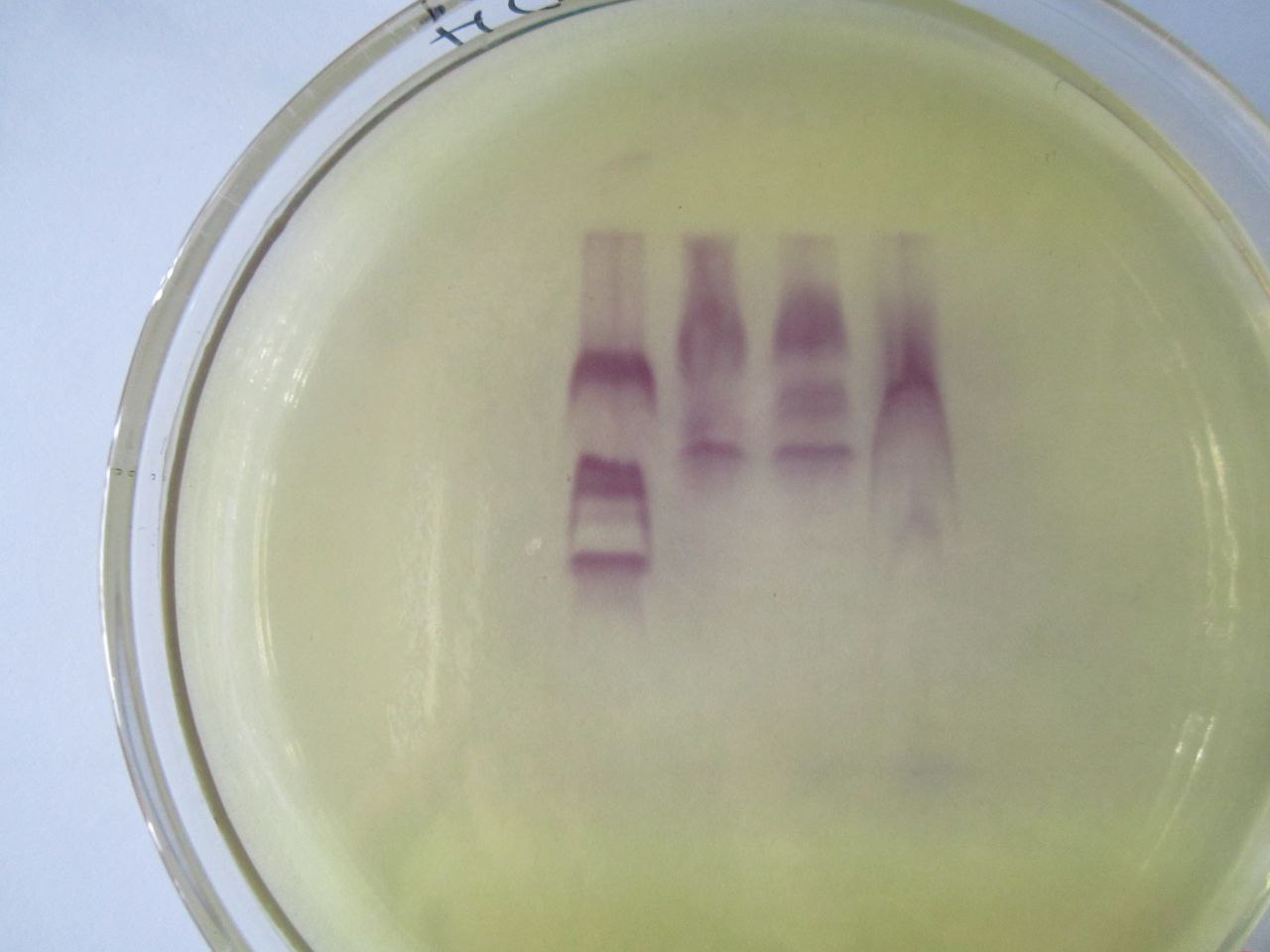


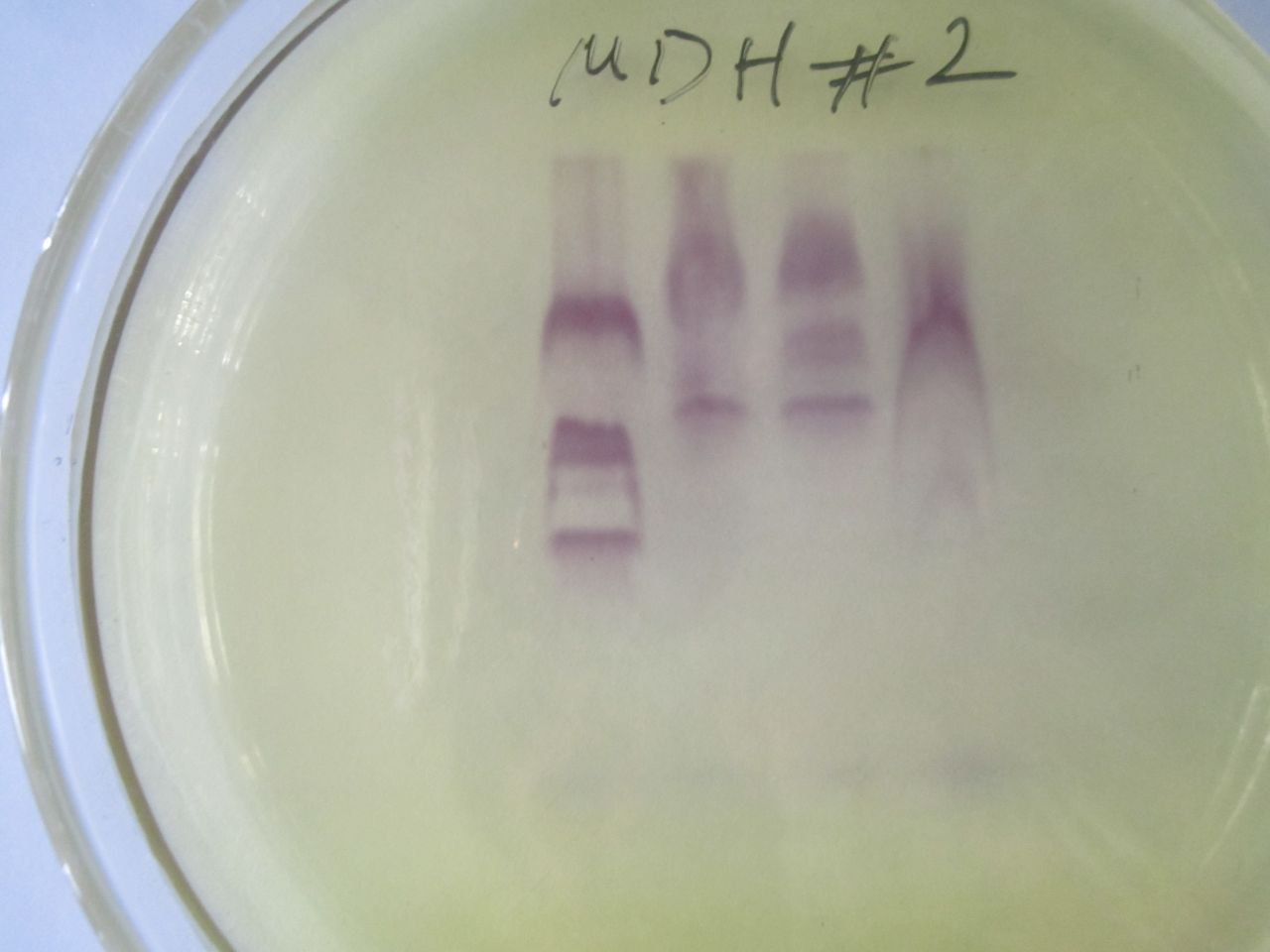


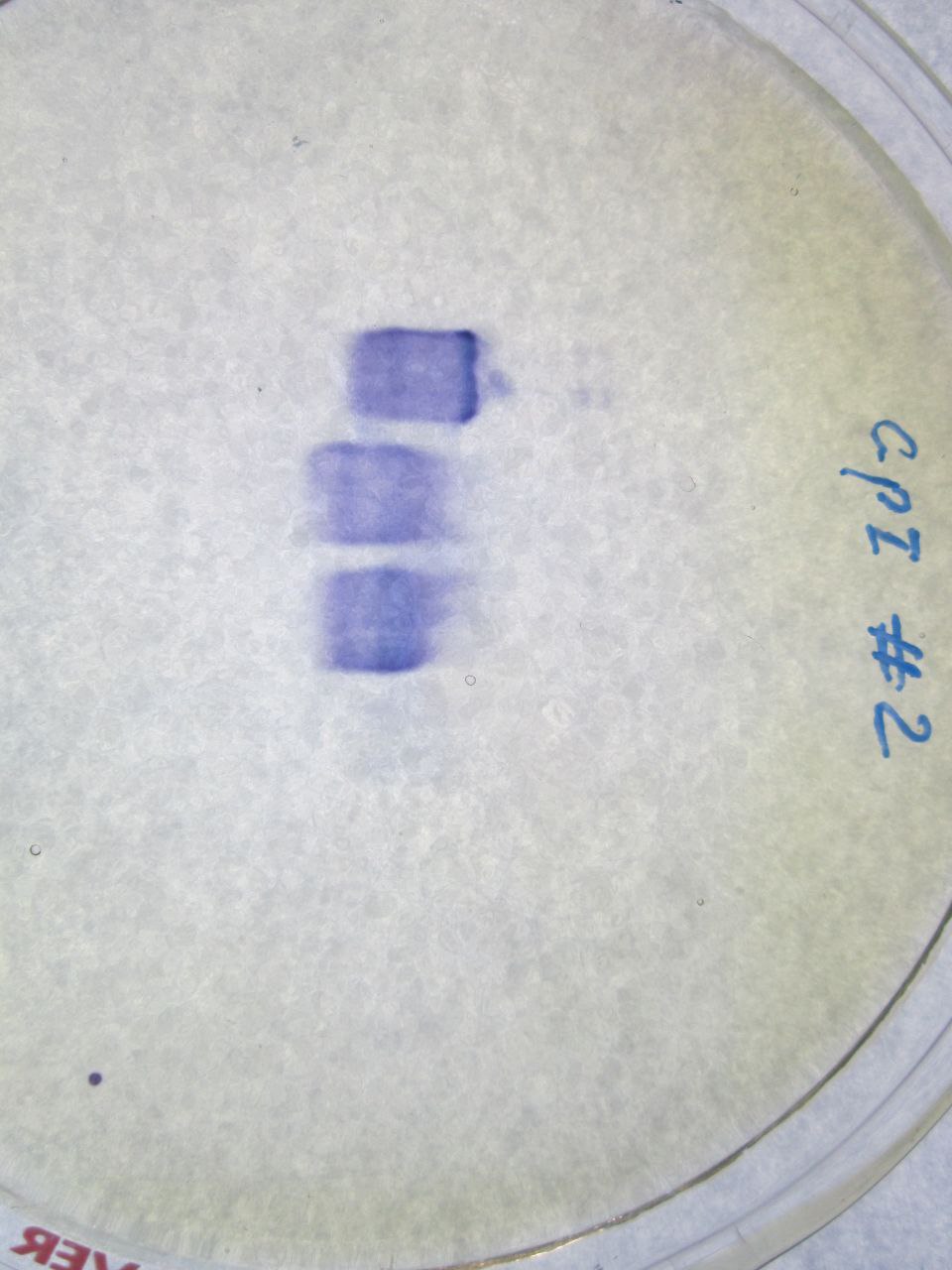


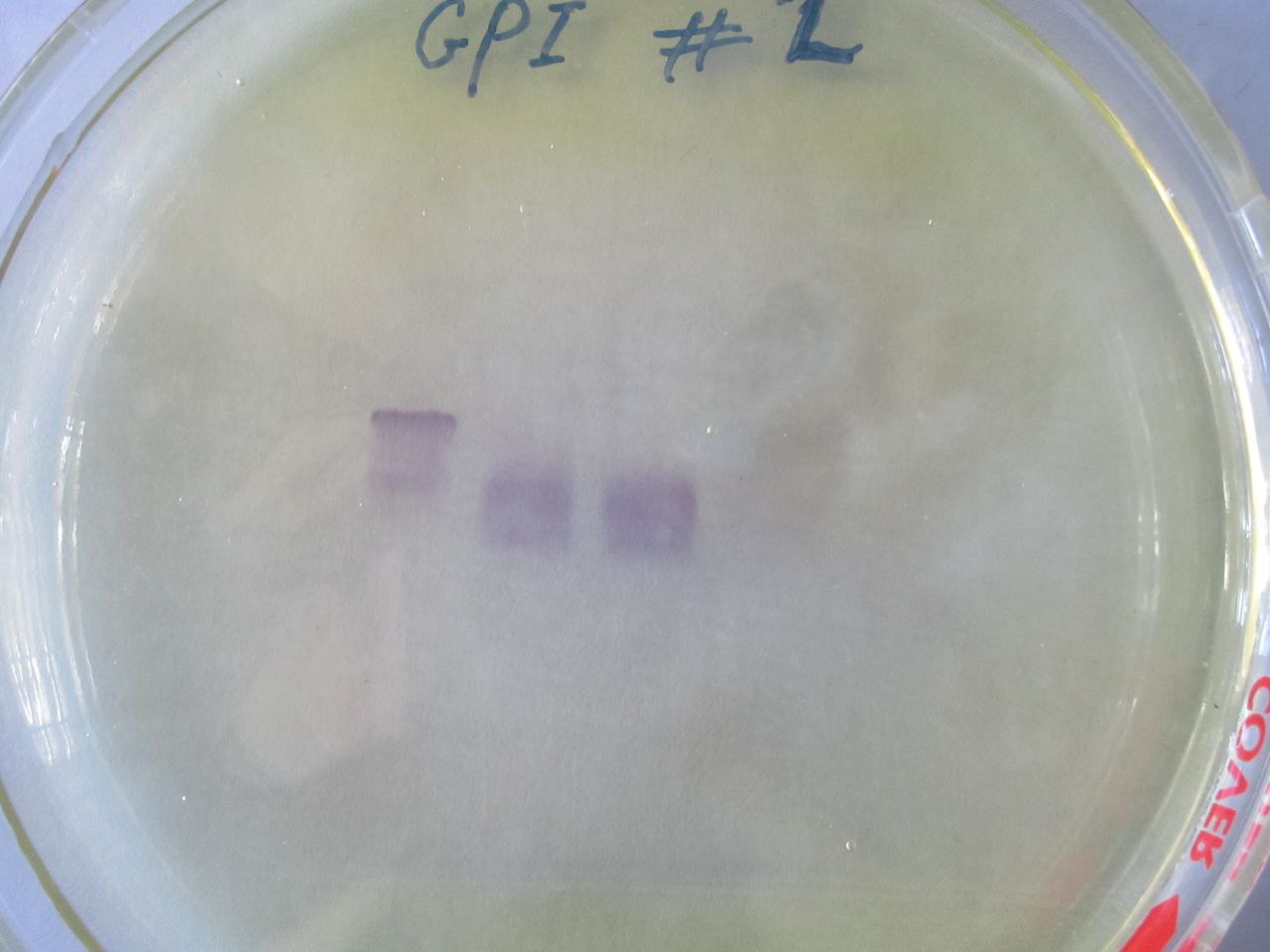


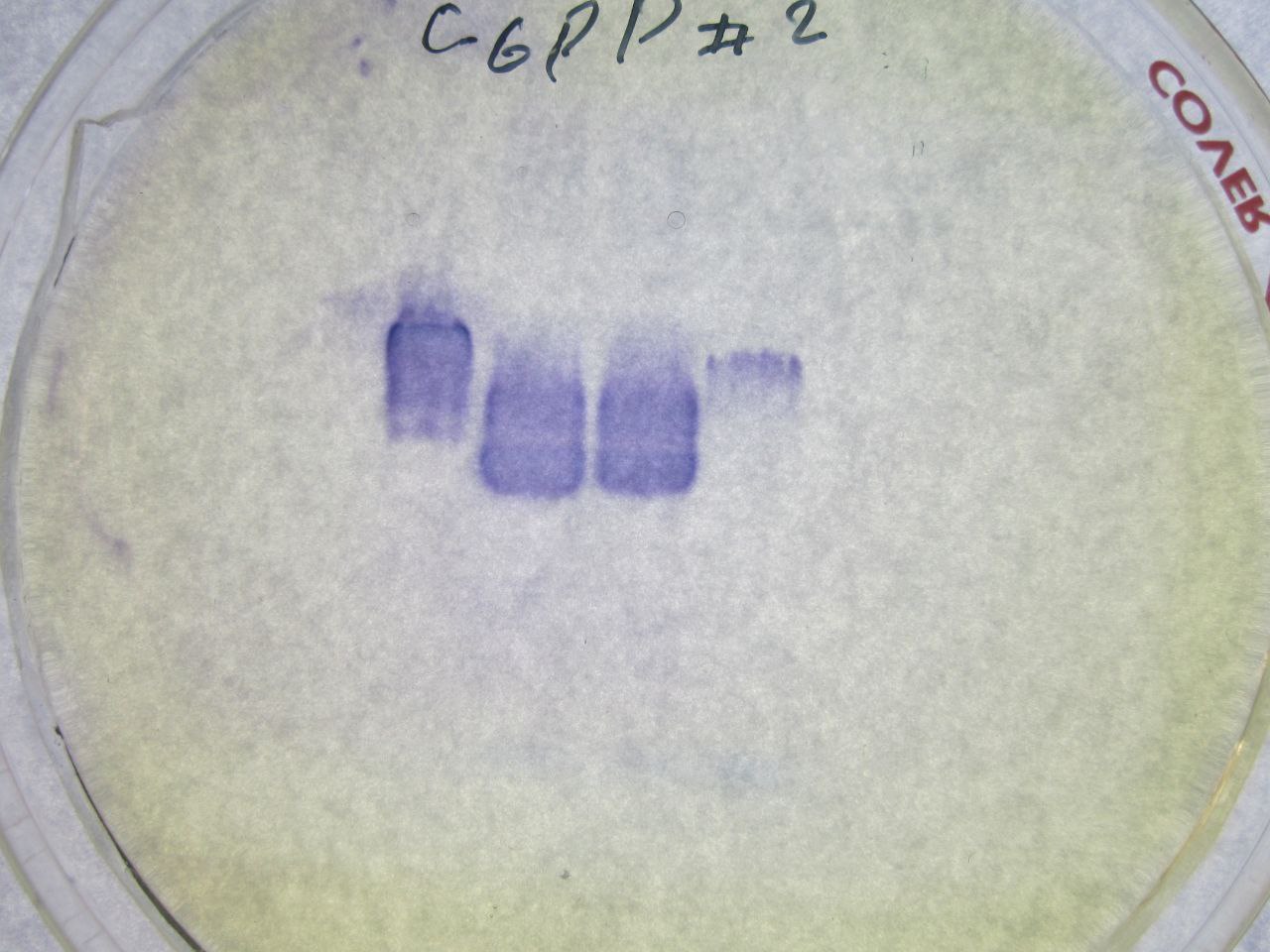


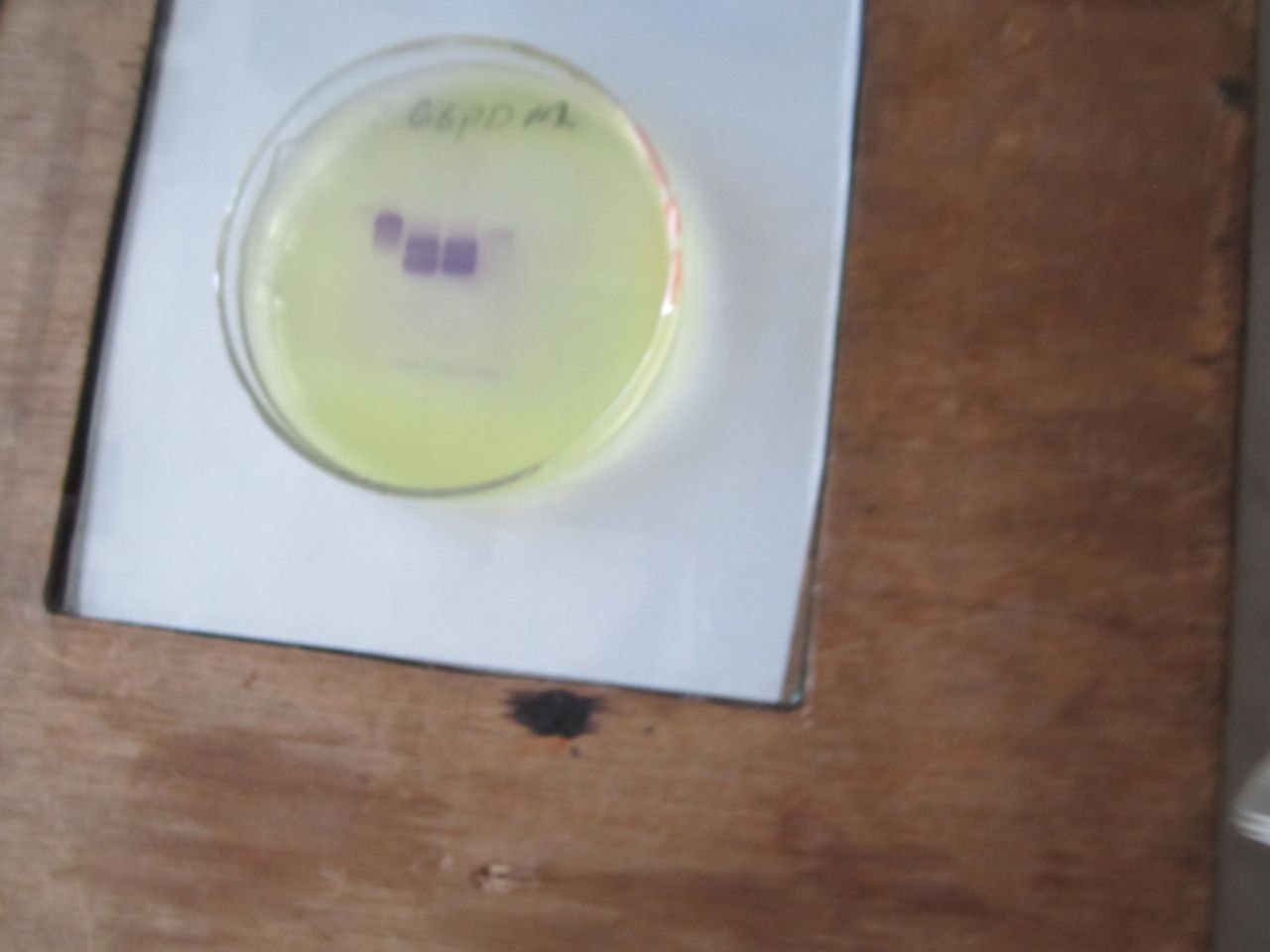


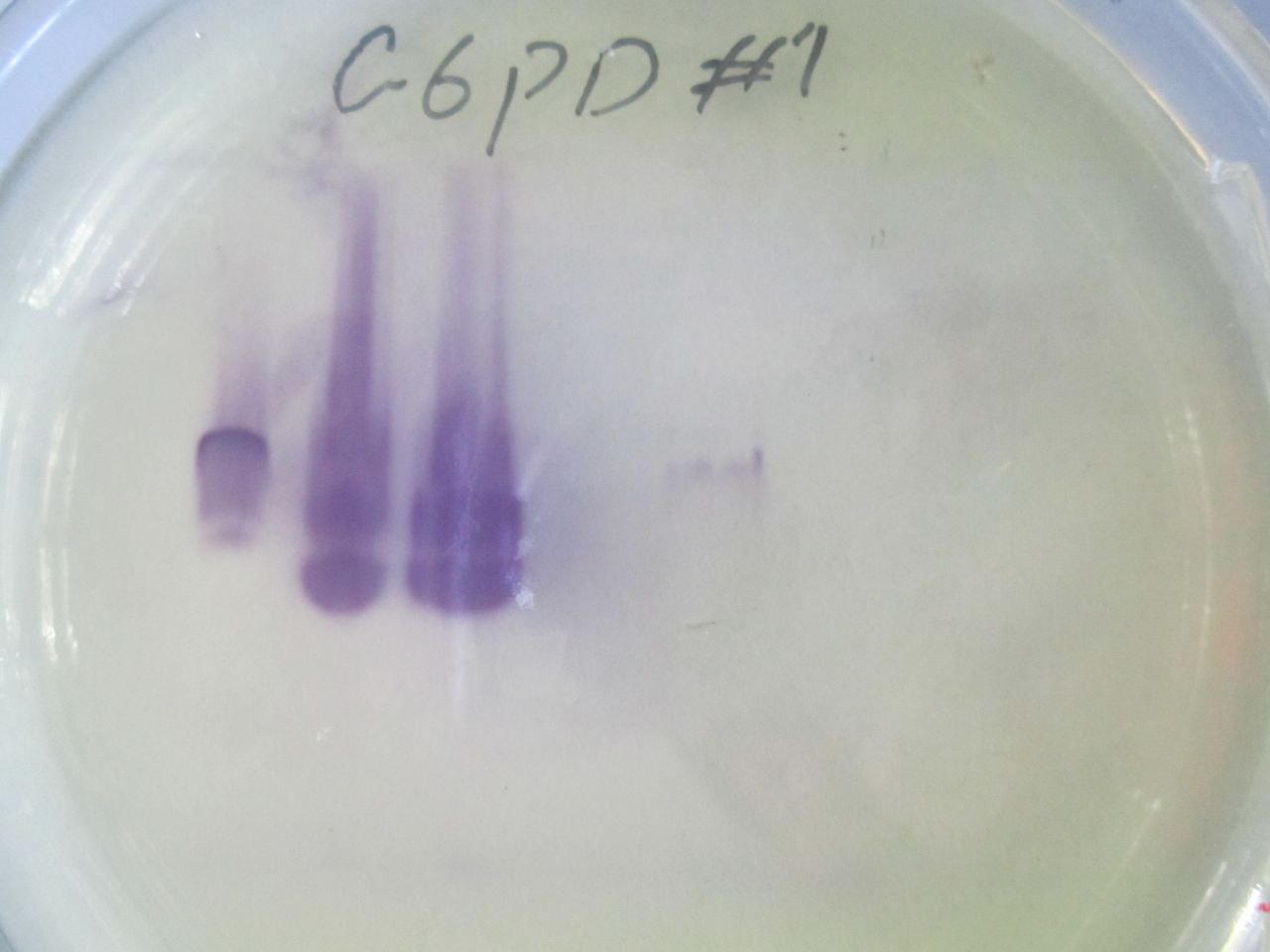

Supplement: Supplementary file 1 — Supplementary Material 1 [file 12879_2024_9069_MOESM1_ESM.docx]
